# Supplementary material for: A comparative driving safety study of mountainous expressway individual tunnel and tunnel group based on eye gaze behavior
Source: PLoS One. 2022 Feb 14;17(2):e0263835. doi: 10.1371/journal.pone.0263835 (PMC8843135; doi:10.1371/journal.pone.0263835)
Supplement: S1 Table — (PDF) [file pone.0263835.s001.pdf]

Short tunnel

Single Short tunnel

(On -step)	cab area	the near road section	the far ahead road section	the vehicle rearview mirrors	tunnel vault and sidewall
cab area	0.894534779	0.064814815	0.040650407	0	0
the near road section	0.018956044	0.935989011	0.041483516	0.003571429	0
the far ahead road section	0.010204082	0.092796093	0.8713588	0	0.025641026
the vehicle rearview mirrors	0	0.25	0	0.75	0
tunnel vault and sidewall	0.125	0	0	0	0.875

(Two-step)	cab area	the near road section	the far ahead road section	the vehicle rearview mirrors	tunnel vault and sidewall
cab area	0.80304742	0.118773378	0.077979202	0.0002	0
the near road section	0.034339711	0.887362656	0.06983077	0.0061	0.002366864
the far ahead road section	0.024571992	0.153510257	0.781630375	0.0001	0.040187377
the vehicle rearview mirrors	0.0018	0.4286	0.0054	0.5642	0
tunnel vault and sidewall	0.220486111	0.013888889	0	0	0.765625

(Stable)	cab area	the near road section	the far ahead road section	the vehicle rearview mirrors	tunnel vault and sidewall
cab area	0.1648	0.4048	0.4007	0.0061	0.0236
the near road section	0.1648	0.4048	0.4007	0.0061	0.0236
the far ahead road section	0.1648	0.4048	0.4007	0.0061	0.0236
the vehicle rearview mirrors	0.1648	0.4048	0.4007	0.0061	0.0236
tunnel vault and sidewall	0.1648	0.4048	0.4007	0.0061	0.0236

Short tunnel in tunnel group

(One- step)	cab area	the near road section	the far ahead road section	the vehicle rearview mirrors	tunnel vault and sidewall
cab area	0.871212121	0.083333333	0.045454545	0	0
the near road section	0.00952381	0.904761905	0.085714286	0	0
the far ahead road section	0.004149378	0.022580645	0.967969482	0.002074689	0.003225806
the vehicle rearview mirrors	0	0	0	0.857142857	0.142857143
tunnel vault and sidewall	0	0.5	0.041666667	0.041666667	0.416666667

(Two- step)	cab area	the near road section	the far ahead road section	the vehicle rearview mirrors	tunnel vault and sidewall
cab area	0.761615873	0.14484127	0.093342857	0.0002	0
the near road section	0.016553289	0.824052372	0.158841344	0	0.000552995
the far ahead road section	0.008065054	0.045071106	0.939654527	0.00415	0.003059313
the vehicle rearview mirrors	0	0	0.0119	0.7511	0.237
tunnel vault and sidewall	0.005111905	0.452380952	0.118707143	0.06465	0.35915

(Stable)	cab area	the near road section	the far ahead road section	the vehicle rearview mirrors	tunnel vault and sidewall
cab area	0.0578	0.1847	0.6891	0.0399	0.0286
the near road section	0.0578	0.1847	0.6891	0.0399	0.0286
the far ahead road section	0.0578	0.1847	0.6891	0.0399	0.0286

the vehicle rearview mirrors	0.0578	0.1847	0.6891	0.0399	0.0286
tunnel vault and sidewall	0.0578	0.1847	0.6891	0.0399	0.0286

Medium tunnel

Single medium tunnel

（One step）	cab area	the near road section	the far ahead road section	the vehicle rearview mirrors	tunnel vault and sidewall
cab area	0.905736509	0.027661643	0.062469616	0	0.004132231
the near road section	0.034966368	0.895170238	0.067471049	0	0.002392344
the far ahead road section	0.01783094	0.047584111	0.928132533	0.001329787	0.005122629
the vehicle rearview mirrors	0	0.027777778	0	0.659722222	0.3125
tunnel vault and sidewall	0	0.033566058	0.033566058	0.026315789	0.906552095

（Two step）	cab area	the near road section	the far ahead road section	the vehicle rearview mirrors	tunnel vault and sidewall
cab area	0.822134178	0.053708658	0.116099698	0.0001	0.007957466
the near road section	0.063107959	0.807204585	0.124416375	0.000275913	0.004995168
the far ahead road section	0.033682531	0.088223966	0.865712921	0.002079634	0.010300948
the vehicle rearview mirrors	0.00033227	0.062918338	0.012775093	0.466445338	0.457528961
tunnel vault and sidewall	0.00191666	0.063053951	0.064375098	0.045160033	0.825494255

（Stable）	cab area	the near road section	the far ahead road section	the vehicle rearview mirrors	tunnel vault and sidewall
cab area	0.1542	0.3320	0.4388	0.0102	0.0648
the near road section	0.1542	0.3320	0.4388	0.0102	0.0648
the far ahead road section	0.1542	0.3320	0.4388	0.0102	0.0648
the vehicle rearview mirrors	0.1542	0.3320	0.4388	0.0102	0.0648
tunnel vault and sidewall	0.1542	0.3320	0.4388	0.0102	0.0648

Medium tunnel in tunnel group

（One step）	cab area	the near road section	the far ahead road section	the vehicle rearview mirrors	tunnel vault and sidewall
cab area	0.889136564	0.045321502	0.059481328	0.004545455	0.001515152
the near road section	0.012759171	0.831166932	0.15281765	0.000730994	0.002525253
the far ahead road section	0.021517379	0.034745817	0.934302786	0.001440129	0.007993888
the vehicle rearview mirrors	0.108333333	0.063888889	0	0.744444444	0.083333333
tunnel vault and sidewall	0.008196721	0.122710623	0.074175824	0	0.794916832

（Two step）	cab area	the near road section	the far ahead road section	the vehicle rearview mirrors	tunnel vault and sidewall
cab area	0.794936041	0.081964092	0.113352921	0.006747116	0.002999828
the near road section	0.026212798	0.70212511	0.26475011	0.001325482	0.005586499
the far ahead road section	0.040109445	0.06383176	0.87927488	0.002717429	0.014066485
the vehicle rearview mirrors	0.157866578	0.107257848	0.02255312	0.581624863	0.130697591
tunnel vault and sidewall	0.019363894	0.196903272	0.129983859	0.000337061	0.653411915

(Stable)	cab area	the near road section	the far ahead road section	the vehicle rearview mirrors	tunnel vault and sidewall
cab area	0.1788	0.2127	0.5430	0.0112	0.0543
the near road section	0.1788	0.2127	0.5430	0.0112	0.0543
the far ahead road section	0.1788	0.2127	0.5430	0.0112	0.0543
the vehicle rearview mirrors	0.1788	0.2127	0.5430	0.0112	0.0543
tunnel vault and sidewall	0.1788	0.2127	0.5430	0.0112	0.0543

Long tunnel

Single long tunnel

(One step)	cab area	the near road section	the far ahead road section	the vehicle rearview mirrors	tunnel vault and sidewall
cab area	0.892600226	0.040142915	0.064539689	0.00035868	0.002358491
the near road section	0.017529051	0.942634406	0.032827857	0.003559823	0.003448862
the far ahead road section	0.012520353	0.068540357	0.904615487	0.004032347	0.010291457
the vehicle rearview mirrors	0.008333333	0.088609307	0.008333333	0.85275974	0.041964286
tunnel vault and sidewall	0.035714286	0.070559896	0.175031009	0	0.718694809

(Two step)	cab area	the near road section	the far ahead road section	the vehicle rearview mirrors	tunnel vault and sidewall
cab area	0.799025	0.0763	0.1191	0.001	0.004575
the near road section	0.032725	0.891875	0.062675	0.0063	0.006425
the far ahead road section	0.024325	0.1252	0.8262	0.007175	0.0171
the vehicle rearview mirrors	0.0179875	0.159775	0.0259	0.7290125	0.067325
tunnel vault and sidewall	0.0524	0.126375	0.273125	0.000925	0.547175

(Stable)	cab area	the near road section	the far ahead road section	the vehicle rearview mirrors	tunnel vault and sidewall
cab area	0.1403	0.4085	0.4030	0.0170	0.0312
the near road section	0.1403	0.4085	0.4030	0.0170	0.0312
the far ahead road section	0.1403	0.4085	0.4030	0.0170	0.0312
the vehicle rearview mirrors	0.1403	0.4085	0.4030	0.0170	0.0312
tunnel vault and sidewall	0.1403	0.4085	0.4030	0.0170	0.0312

Long tunnel in tunnel group

(One step)	cab area	the near road section	the far ahead road section	the vehicle rearview mirrors	tunnel vault and sidewall
cab area	0.890981372	0.020195416	0.08410391	0.00259214	0.002127163
the near road section	0.033081334	0.858958039	0.078391284	0.008583366	0.020985978
the far ahead road section	0.017632408	0.020015937	0.954765329	0.002116116	0.00547021
the vehicle rearview mirrors	0	0.069480057	0.020535359	0.737172105	0.172812434
tunnel vault and sidewall	0.008821699	0.035260214	0.072019686	0.010694816	0.873203584

(Two step)	cab area	the near road section	the far ahead road section	the vehicle rearview mirrors	tunnel vault and sidewall
cab area	0.796235927	0.037983171	0.156391389	0.00449743	0.004892083

the near road section	0.057833728	0.745545828	0.14470802	0.014707333	0.037205091
the far ahead road section	0.033108865	0.03746309	0.915027299	0.00383186	0.010568886
the vehicle rearview mirrors	0.002999501	0.115668787	0.055535069	0.598650507	0.227146135
tunnel vault and sidewall	0.017398476	0.063755293	0.133984898	0.016312829	0.768548503

(Stable)	cab area	the near road section	the far ahead road section	the vehicle rearview mirrors	tunnel vault and sidewall
cab area	0.1427	0.1786	0.6034	0.0191	0.0562
the near road section	0.1427	0.1786	0.6034	0.0191	0.0562
the far ahead road section	0.1427	0.1786	0.6034	0.0191	0.0562
the vehicle rearview mirrors	0.1427	0.1786	0.6034	0.0191	0.0562
tunnel vault and sidewall	0.1427	0.1786	0.6034	0.0191	0.0562

Extra-long tunnel

Single extra-long tunnel

(One step)	cab area	the near road section	the far ahead road section	the vehicle rearview mirrors	tunnel vault and sidewall
cab area	0.885964718	0.065525252	0.040029163	0.002786215	0.005694652
the near road section	0.02797182	0.947376995	0.018296885	0.002411102	0.003943198
the far ahead road section	0.015110875	0.061922171	0.912225585	0.002405846	0.008335522
the vehicle rearview mirrors	0.021712407	0.054467482	0.006060606	0.790506545	0.12725296
tunnel vault and sidewall	0.006697819	0.14401164	0.046255996	0.033722864	0.769311681

(Two step)	cab area	the near road section	the far ahead road section	the vehicle rearview mirrors	tunnel vault and sidewall
cab area	0.787322963	0.122917337	0.073629306	0.005164994	0.010965399
the near road section	0.051009428	0.901224831	0.035556192	0.004611409	0.00759814
the far ahead road section	0.0285959	0.116197369	0.83511031	0.004512552	0.01558387
the vehicle rearview mirrors	0.038560397	0.110298958	0.015631587	0.672614843	0.162894215
tunnel vault and sidewall	0.015172969	0.237982488	0.082760516	0.046452623	0.617631405

(Stable)	cab area	the near road section	the far ahead road section	the vehicle rearview mirrors	tunnel vault and sidewall
cab area	0.1317	0.4883	0.2953	0.0205	0.0642
the near road section	0.1317	0.4883	0.2953	0.0205	0.0642
the far ahead road section	0.1317	0.4883	0.2953	0.0205	0.0642
the vehicle rearview mirrors	0.1317	0.4883	0.2953	0.0205	0.0642
tunnel vault and sidewall	0.1317	0.4883	0.2953	0.0205	0.0642

Extra-long tunnel in tunnel group

(One step)	cab area	the near road section	the far ahead road section	the vehicle rearview mirrors	tunnel vault and sidewall
cab area	0.89292325	0.060276252	0.04423319	0.00139178	0.001175528
the near road section	0.014404261	0.938935155	0.03954893	0.003034623	0.004077031
the far ahead road section	0.013169278	0.062126754	0.914341357	0.000940221	0.00942239

the vehicle rearview mirrors	0	0.177725942	0.006938776	0.699599506	0.115735776
tunnel vault and sidewall	0.019630432	0.129698118	0.126142989	0.010169492	0.71435897

（Two step）	cab area	the near road section	the far ahead road section	the vehicle rearview mirrors	tunnel vault and sidewall
cab area	0.799281024	0.112452616	0.083326274	0.00250952	0.002430567
the near road section	0.027011324	0.88543414	0.075050276	0.005435891	0.007068369
the far ahead road section	0.024760674	0.116010878	0.841959126	0.002120489	0.015148833
the vehicle rearview mirrors	0.0031045	0.179036772	0.024145059	0.702826037	0.090887632
tunnel vault and sidewall	0.03487838	0.217742238	0.216216202	0.015783791	0.515379388

（Stable）	cab area	the near road section	the far ahead road section	the vehicle rearview mirrors	tunnel vault and sidewall
cab area	0.1237	0.3904	0.4415	0.0125	0.0319
the near road section	0.1237	0.3904	0.4415	0.0125	0.0319
the far ahead road section	0.1237	0.3904	0.4415	0.0125	0.0319
the vehicle rearview mirrors	0.1237	0.3904	0.4415	0.0125	0.0319
tunnel vault and sidewall	0.1237	0.3904	0.4415	0.0125	0.0319
