# Supplementary material for: A comparative driving safety study of mountainous expressway individual tunnel and tunnel group based on eye gaze behavior
Source: PLoS One. 2022 Feb 14;17(2):e0263835. doi: 10.1371/journal.pone.0263835 (PMC8843135; doi:10.1371/journal.pone.0263835)
Supplement: Attachment — Submitted filename: PONE-D-21-35527 marked.pdf [file pone.0263835.s002.pdf]

PLOS ONE

A Comparative Driving Safety Study of Mountainous Expressway Individual Tunnel and Tunnel Group Based on Eye Gaze Behavior

--Manuscript Draft--

**Manuscript Number:**	PONE-D-21-35527
**Article Type:**	Research Article
**Full Title:**	A Comparative Driving Safety Study of Mountainous Expressway Individual Tunnel and Tunnel Group Based on Eye Gaze Behavior
**Short Title:**	Study of Mountainous Expressway Individual Tunnel and Tunnel Group Based on Eye Gaze Behavior
**Corresponding Author:**	An Huang<br>Chongqing Jiaotong University<br>China, CHINA
**Keywords:**	Mountainous Expressway; Tunnel Group; Individual Tunnel; Eye-gaze Behavior; Markov Theory
**Abstract:**	The traffic environment of a tunnel group is more complex than that of a single tunnel, which increases the driving risk. The eye gaze behavior of drivers can be used to evaluate driving safety and comfort. To analyze the fixation characteristics of drivers in a single tunnel and tunnel group of mountainous expressways, an actual vehicle test is conducted. The test area has a total length of 160 km and 38 tunnels, including 8 tunnel groups and 16 single tunnels. In the test, the difference in the gaze time of seven drivers between single tunnels and tunnel groups is compared. The k-means method is used to cluster driver's gaze points dynamically. Based on the Markov theory, the attributes related to gaze transfer are obtained. The results show that when tunnels are of short or medium length, there is no significant difference in the gaze time and gaze point transfer between the tunnel group and a single tunnel. In contrast, when tunnels have long or extra-long length, the repeated fixation probability and the two-step transition probability of looking back of a driver in a tunnel group are higher than those in a single tunnel. The design and management method of a single tunnel cannot be directly used, especially for extra-long tunnels located at the back of a tunnel group with a long upstream tunnel length and a short interval distance from the upstream tunnel. Therefore, it is necessary to focus on the design and management methods of tunnel groups.
**Order of Authors:**	An Huang
	Ting Shang
	Hongjiao Qi
	Tangzhi Liu
**Additional Information:**	
**Question**	**Response**
**Financial Disclosure**<br><br>Enter a financial disclosure statement that describes the sources of funding for the work included in this submission. Review the submission guidelines for detailed requirements. View published research articles from *PLOS ONE* for specific examples.<br><br>This statement is required for submission and **will appear in the published article** if	Yes.<br>1.This work was supported by the Science and Technology Research Program of Chongqing Municipal Education Commission (Grant No. KJQN201900722) and the Science and Technology Bureau Foundation and Frontier Project of Chongqing (Grant No. cstc2019jcyj-msxmX0695). We thank LetPub (www.letpub.com) for its linguistic assistance during the preparation of this manuscript.<br>2.This work was supported by the Science and Technology Research Program of Chongqing Municipal Education Commission (Grant No. KJQN201900722) and the Science and Technology Bureau Foundation and Frontier Project of Chongqing (Grant No. cstc2019jcyj-msxmX0695).<br>3.National Natural Science Foundation of China(Grant No.52172341)

Powered by Editorial Manager® and ProduXion Manager® from Aries Systems Corporation

the submission is accepted. Please make sure it is accurate.<br><br>**Unfunded studies**<br>Enter: *The author(s) received no specific funding for this work.*<br><br>**Funded studies**<br>Enter a statement with the following details:<br>• Initials of the authors who received each award<br>• Grant numbers awarded to each author<br>• The full name of each funder<br>• URL of each funder website<br>• Did the sponsors or funders play any role in the study design, data collection and analysis, decision to publish, or preparation of the manuscript?<br>• **NO** - Include this sentence at the end of your statement: *The funders had no role in study design, data collection and analysis, decision to publish, or preparation of the manuscript.*<br>• **YES** - Specify the role(s) played.<br><br>* typeset	
**Competing Interests**<br><br>Use the instructions below to enter a competing interest statement for this submission. On behalf of all authors, disclose any competing interests that could be perceived to bias this work—acknowledging all financial support and any other relevant financial or non-financial competing interests.<br><br>This statement is required for submission and **will appear in the published article** if the submission is accepted. Please make sure it is accurate and that any funding sources listed in your Funding Information later in the submission form are also declared in your Financial Disclosure statement.<br><br>View published research articles from *PLOS ONE* for specific examples.	NO authors have competing interests.

**NO authors have competing interests**<br><br>Enter: *The authors have declared that no competing interests exist.*<br><br>**Authors with competing interests**<br><br>Enter competing interest details beginning with this statement:<br><br>*I have read the journal's policy and the authors of this manuscript have the following competing interests: [insert competing interests here]*<br><br>* typeset	
**Ethics Statement**<br><br>Enter an ethics statement for this submission. This statement is required if the study involved:<br><br>• Human participants<br>• Human specimens or tissue<br>• Vertebrate animals or cephalopods<br>• Vertebrate embryos or tissues<br>• Field research<br><br>Write "N/A" if the submission does not require an ethics statement.<br><br>General guidance is provided below. Consult the submission guidelines for detailed instructions. **Make sure that all information entered here is included in the Methods section of the manuscript.**	N/A

**Format for specific study types**<br><br>**Human Subject Research (involving human participants and/or tissue)**<br>• Give the name of the institutional review board or ethics committee that approved the study<br>• Include the approval number and/or a statement indicating approval of this research<br>• Indicate the form of consent obtained (written/oral) or the reason that consent was not obtained (e.g. the data were analyzed anonymously)<br><br>**Animal Research (involving vertebrate animals, embryos or tissues)**<br>• Provide the name of the Institutional Animal Care and Use Committee (IACUC) or other relevant ethics board that reviewed the study protocol, and indicate whether they approved this research or granted a formal waiver of ethical approval<br>• Include an approval number if one was obtained<br>• If the study involved *non-human primates*, add *additional details* about animal welfare and steps taken to ameliorate suffering<br>• If anesthesia, euthanasia, or any kind of animal sacrifice is part of the study, include briefly which substances and/or methods were applied<br><br>**Field Research**<br><br>Include the following details if this study involves the collection of plant, animal, or other materials from a natural setting:<br>• Field permit number<br>• Name of the institution or relevant body that granted permission	
**Data Availability**<br><br>Authors are required to make all data underlying the findings described fully available, without restriction, and from the time of publication. PLOS allows rare exceptions to address legal and ethical concerns. See the PLOS Data Policy and FAQ for detailed information.	Yes - all data are fully available without restriction

A Data Availability Statement describing where the data can be found is required at submission. Your answers to this question constitute the Data Availability Statement and **will be published in the article**, if accepted.<br><br>**Important:** Stating 'data available on request from the author' is not sufficient. If your data are only available upon request, select 'No' for the first question and explain your exceptional situation in the text box.<br><br>Do the authors confirm that all data underlying the findings described in their manuscript are fully available without restriction?	
**Describe where the data may be found in full sentences. If you are copying our sample text, replace any instances of XXX with the appropriate details.**<br><br>• If the data are **held or will be held in a public repository**, include URLs, accession numbers or DOIs. If this information will only be available after acceptance, indicate this by ticking the box below. For example: *All XXX files are available from the XXX database (accession number(s) XXX, XXX.)*.<br>• If the data are all contained **within the manuscript and/or Supporting Information files**, enter the following: *All relevant data are within the manuscript and its Supporting Information files.*<br>• If neither of these applies but you are able to provide **details of access elsewhere**, with or without limitations, please do so. For example:<br><br>*Data cannot be shared publicly because of [XXX]. Data are available from the XXX Institutional Data Access / Ethics Committee (contact via XXX) for researchers who meet the criteria for access to confidential data.*<br><br>*The data underlying the results presented in the study are available from (include the name of the third party*	All relevant data are within the manuscript and its Supporting Information files.

and contact information or URL).<br>• This text is appropriate if the data are owned by a third party and authors do not have permission to share the data.<br><br>* typeset	
Additional data availability information:	Tick here if the URLs/accession numbers/DOIs will be available only after acceptance of the manuscript for publication so that we can ensure their inclusion before publication.

Manuscript Click here to access/download;Manuscript;Manuscript.docx

JOURNAL OF ADVANCED TRANSPORTATION

A Comparative Driving Safety Study of Mountainous Expressway Individual Tunnel and Tunnel Group Based on Eye Gaze Behavior

Ting M. I. Shang,[1] Hongjiao A. Qi,[1] An B. Huang[1] and Tangzhi C. Liu[1]

[1] School of Traffic & Transportation, Chongqing Jiaotong University, Chongqing 400074, China.

Correspondence should be addressed to An B. Huang;622200950083@mails.cqjtu.edu.cn

Abstract

The traffic environment of a tunnel group is more complex than that of a single tunnel, which increases the driving risk. The eye gaze behavior of drivers can be used to evaluate driving safety and comfort. To analyze the fixation characteristics of drivers in a single tunnel and tunnel group of mountainous expressways, an actual vehicle test is conducted. The test area has a total length of 160 km and 38 tunnels, including 8 tunnel groups and 16 single tunnels. In the test, the difference in the gaze time of seven drivers between single tunnels and tunnel groups is compared. The k-means method is used to cluster driver's gaze points dynamically. Based on the Markov theory, the attributes related to gaze transfer are obtained. The results show that when tunnels are of short or medium length, there is no significant difference in the gaze time and gaze point transfer between the tunnel group and a single tunnel. In contrast, when tunnels have long or extra-long length, the repeated fixation probability and the two-step transition probability of looking back of a driver in a tunnel group are higher than those in a single tunnel. The design and management method of a single tunnel cannot be directly used, especially for extra-long tunnels located at the back of a tunnel group with a long upstream tunnel length and a short interval distance from the upstream tunnel. Therefore, it is necessary to focus on the design and management methods of tunnel groups.

Keywords: Mountainous Expressway, Tunnel Group, Individual Tunnel, Eye-gaze Behavior, Markov Theory

1.Introduction

A tunnel has obvious advantages in breaking through the geographical barrier and shortening the journey. However, due to a single closed space, poor line of sight, and sharp change in brightness at the entrance and exit of a tunnel, there are problems of high traffic intensity and accident rate. With the development of expressways to remote mountainous areas, the number and scale of tunnels have increased. Currently, the largest tunnel group in the world includes 136 tunnels and is on the Xihan Expressway in Shanxi Province, China. Due to a large number of tunnels and a short distance between them, the light environment at the entrance and exit of a tunnel group changes dramatically, which puts an excessive load on a driver's vision and makes the traffic safety risk more prominent [1]. Driving is a complex

task, and different needs compete for a driver's attention. Suitable design of a tunnel group is favorable to driver's cognition of key information and relieves visual load. This study compares differences in the driver's gaze behavior between a single tunnel and a tunnel group and defines the high-risk factors affecting the operation of a tunnel group so as to ensure driving safety.

Tunnel groups are very common in mountain expressways. A tunnel group can constitute about 80% of an expressway, whereas the roadbed section can constitute only about 20% of an expressway. There are large differences between a tunnel group and a single tunnel in the number of tunnels and the length of distance between two tunnels, which causes a driver to experience the alternation of black and white hole effects in a short time. The cumulative visual superposition effect is obvious, which causes an excessive visual load to drivers and can easily cause traffic accidents due to uncontrolled operation. However, the current Chinese standards [2-5] and the Unified Traffic Control Equipment Manual (MUTCD) 2009 guidelines do not define the linear design specifications for tunnel groups. The existing tunnel groups are designed according to the specification requirements of a single tunnel. Therefore, how to effectively design a mountain expressway tunnel group and ensure traffic safety has become a challenge. The driver's gaze behavior can properly express the visual information processing, so the change in the driver's gaze behavior can be used to evaluate the safety and comfort of an expressway tunnel group. In this study, the difference in driver's fixation duration between a single tunnel and a tunnel group is analyzed. The Markov theory is used to determine the fixation transfer regularity and stationary distribution characteristics, and the high-risk factors affecting the operation of a tunnel group are defined to ensure driving safety.

To analyze the safety of a single tunnel and a tunnel group in mountainous expressways from the perspective of drivers, 38 tunnels, including 8 tunnel groups and 16 single tunnels, from Shuijiang to Qianjiang section are used as a research object to perform a real vehicle test and to compare differences in the drivers' fixation durations between single tunnels and tunnel groups. The k-means method is used to cluster driver's gaze points dynamically and to analyze the distribution of gaze points in different interest areas. The Markov chain, including one- and two-step gaze transition probabilities and area gaze probabilities, is used to determine the gaze transfer regularity and stationary distribution and to compare the difference between the single tunnel and tunnel group in the driver's eye gaze characteristics. The findings of this work can provide a useful basis for tunnel group design in mountainous expressways.

2.LITERATURE REVIEW

2.1 Driving Safety in Single Tunnel and Driver's Visual Characteristics

Driving through a tunnel mainly relies on visual perception and access to traffic information, so the driver's eye gaze behavior can be used to determine whether the tunnel driving environment is scientific and reasonable and whether it meets the physiological needs of drivers, and this topic has drawn widespread attention in scientific research. Verwey [6] found that the closer to the tunnel a driver is, the lower the number of human gazes and the scanning range will be. Qi [7] and Yang [8] tested the driver's eye movement data in the tunnel environment. Ye [9] conducted a driving simulation test, and the pupil diameter and

the sight deviation time were analyzed. He (sb' family name) [10] found that at the entrance and exit of the tunnel, drivers experienced the process of alternating cycle mutation of light environment and rapid conversion of visual light and dark adaptation. In addition, Du [11] considered the target sign's location of a driver's visual field and established the tunnel sign's visual cognition probabilistic model based on driver's eye-movement characteristics. Shang [12] employed the Markov chain to analyze the driver's eye movement characteristics to evaluate the effect of separate guide signs in a tunnel. Bai [13] analyzed the physical and psychological effects of drivers on traffic sign recognition. Pan (14) and Wang [15] studied the relationship between the eye movement index and horizontal alignment at the entrance of a highway tunnel. Tang [16] and Huang [17] researched the colors of pavement and anti-slipping layer on driving safety at the tunnel entrance based on the eye movement indexes.

However, a single tunnel is relatively different from a tunnel group in terms of length, geometrical design, and illumination characteristics. In particular, tunnel groups on mountainous highways are more affected by the black- and white-hole effects and have a larger impact on a driver's vision and driving characteristics than a single tunnel. Therefore, it is necessary to compare driving safety of a single tunnel and a tunnel group based on the eye behavior.

2.2 Driving Safety in Tunnel Group

Tunnel groups contribute local economic and social development to the infinite vitality, but there are certain challenges in determining accident-prone tunnel groups. There are large differences between tunnel group and a single tunnel, so the characteristics of traffic accidents in them are also different. Wang [18] studied the characteristics of traffic crashes in the freeway tunnel groups using a five-zone analytic approach. Xiong [19] analyzed the accident rate of the tunnel group sections of an expressway from the aspects of road alignment and the anti-sliding ability of the road surface. He (sb' family name) [20] studied the distribution of driver's attention in a mountain expressway's tunnel group sections. Yan [21,22] analyzed the influence of the drivers' visual changes in highway tunnel groups on traffic safety. Using advanced monitoring and rescue technologies can effectively reduce the probability of tunnel groups accidents and reduce the consequences of accidents. Li [23] presented a cooperative control technology under typical emergencies in freeway tunnel groups, which was proposed to achieve coordinated cooperation between lane indicators, video monitors, and other electromechanical facilities. Guo [24] analyzed the intelligent linkage control technology to prevent traffic accidents from becoming worse and the secondary disasters from happening. Jiang [25] designed an intelligent ventilation system, which expanded the application of intelligent tunnel ventilation control systems and could provide design references to other linkage ventilation control systems of tunnel groups.

Research on traffic safety of a tunnel group has been mainly focused on analyses of accident characteristics, lighting, driving eye movement characteristics, and emergency rescue. However, an ordinary highway tunnel and an expressway tunnel group are relatively different in the speed limit, traffic composition, and lighting. In addition, there has been no comparative analysis between a tunnel group and a single tunnel, so it is impossible to know the difference between them in a driver's visual perception.

2.3. Markov Chain Application in Visual Gaze Analysis

There has been a pressing need for quantitative comparisons of eye movement metrics [26]. Many quantitative comparison methods have been developed for two leading eye movement metrics, the scanpaths and the Markov chain, whose next state depends only on the current state and not on the past states. It works by quantizing the complexity within a system into a set of discrete states and a transition relationship between them [27]. In the eye-tracking study [28], Markov Chain models have been applied in the development of the probabilistic algorithm for gaze trajectory prediction. Chen [29] used Markov modeling to realize the eye tracking to obtain insight into a tv customer's experience. Recently, a study on the BT Ireland Innovation Centre (BTIIC) in Belfast was conducted [30], where an eye tracker was employed to evaluate customer experience of the BT Player, which is a video-on-demand application offered by the BT in the UK as part of the IPTV service, BT TV. Chatterjee [31] assumed eye movement parameters were random variables generated by an underlying stochastic process and modeled the fixation sequences of participants using a hidden Markov model. Mauricio [32] studied how elements impacting the driver's visual behavior could provide an insight into the degree to which a vehicle's user interface influenced the attentional focus. Reimer [33] compared visual demand during the baseline driving and the driving while using voice or manual inputs to place calls with Chevrolet MyLink, and using the hidden Markov modeling to characterize the similarity of glance sequences during the two driving types. Markov chain theory has often been used to analyze drivers' visual transfer characteristics. The Markov chain can also be used to study the driver's gaze transition characteristics in a tunnel group.

Plenty of research has been conducted on tunnel traffic safety and operation management, and many results of theoretical and practical significance in scientific research have been obtained. However, there has been no definition of tunnel groups, and there has been little research on tunnel groups of mountainous expressways. At the same time, the existing relevant design specifications of a tunnel group have been mainly based on a single tunnel's technology and experience. However, the number, length, and spacing of tunnels in tunnel groups differ from those of single tunnels. In addition, there has been a lack of in-depth research on influencing mechanisms of driving safety and visual characteristics of drivers in tunnel group sections.

To analyze differences in traffic safety between single tunnels and tunnel groups with different lengths, this paper conducts real vehicle tests on the single tunnel and tunnel group using an eye tracker and obtains gaze transitions based on the area division and Markov theory.

3. METHODS

To reflect changes in the driver's eye movement during the driving process in a tunnel, this paper presents real vehicle tests on 38 tunnels performed by seven drivers. The eye tracker, non-contact multifunctional speedometer, illuminance meter, and other test equipment were used to collect the test data of the driver's fixation point, driving speed, and illuminance value in and out of the tunnel. The k-means method was used to dynamically cluster the driver's fixation point and to analyze the distribution law of a fixation point in different interest areas. Based on the area division and Markov theory, attributes related to gaze transition, including one- and two-step gaze transition probabilities and area gaze

probabilities, were obtained, and differences in the eye gaze behavior between the single tunnel and tunnel group were analyzed.

3.1 Single Tunnel and Tunnel Group Experiments

The Shuijiang to Qianjiang section of Chongqing-Xiangyang expressway in China is 160 km long and includes two-way four-lane highways. It passes through the mountainous area and has 38 tunnels; it belongs to the typical high tunnel ratio section of the mountainous expressway. Since there is no accurate definition of a tunnel group at present, considering the specification and existing research [10,15,23-24,34–35], in this study, a group of two or more tunnels with a spacing of less than 500 m is considered a tunnel group. Details of different tunnel type in the tunnel groups are shown in Table. 1. Accordingly, the test section is divided into 8 tunnel groups and 16 single tunnels (excluding tunnel groups) as shown in Figure 1. The speed limit in the tunnel is in the range of 60–80 km/h, which is the speed limit by specification, and the average longitudinal slope is less than 2%. There is little difference in the operating environment in the test section.

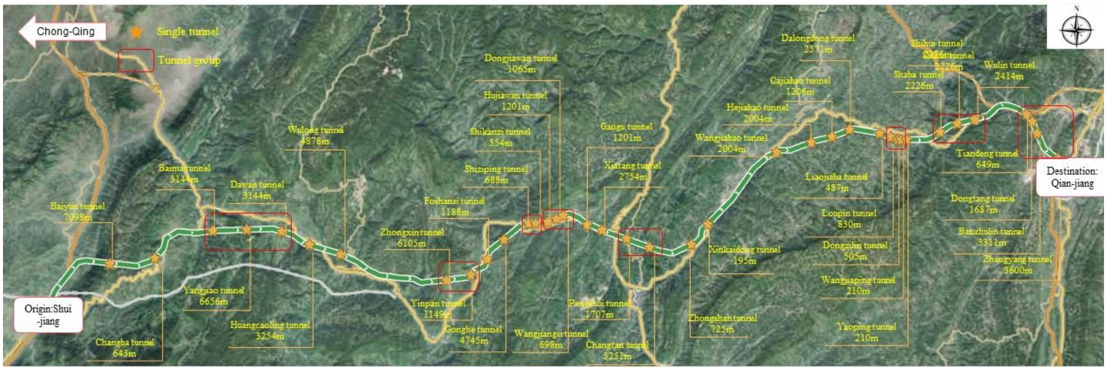

Figure 1: Actual vehicle test route

Table 1: Details of Different tTunnel Type in Tunnel Groups.

Tunnel type	Tunnel name	Tunnel group name	Tunnel type and spacing
Short tunnel	“Shikanzi” tunnel	“Shiziping” tunnel - **“Shikanzi” tunnel** (1#Tunnel Group)	Medium tunnel (688 m) - Spacing (493 m) - **Short tunnel (354 m)**
	“Wangjiaping” tunnel	“Louping” tunnel - “Tongzilin” tunnel - **“Wangjiaping” tunnel**	Medium tunnel (830 m) - Spacing (365 m) - Medium tunnel (505 m) - Spacing (110 m) - **Short tunnel (210 m)**
	“Zhengyang2#” tunnel	“Zhengyang1#” tunnel - **“Zhengyang2# ” tunnel**	Extra-long tunnel (3285 m) - Spacing (47 m) - **Short tunnel (308 m)**
Medium tunnel	“Shiziping” tunnel	**“Shiziping”** tunnel - “Shikanzi” tunnel	**Medium tunnel (688 m)** - Spacing (493 m) - Short tunnel (354 m)
	“Wangjiangsi” tunnel	“Hujiawan” tunnel - “Dongjiawan” tunnel - **“Wangjiangsi” tunnel**	Long tunnel (688 m) - Spacing (79 m) – Long tunnel (1065 m) - Spacing (315 m)- **Medium tunnel (698 m)**

	"Louping" tunnel	**"Louping" tunnel** - "Tongzilin" tunnel - "Wangjiaping" tunnel	**Medium tunnel (830 m)** - Spacing (365 m) - Medium tunnel (354 m) - Spacing (110 m) – Short tunnel (210 m)
	"Tongzilin" tunnel	"Louping" tunnel - **"Tongzilin" tunnel** - "Wangjiaping" tunnel	Medium tunnel (830 m) - Spacing (365 m) - **Medium tunnel (354 m)** - Spacing (110 m) - Short tunnel (210m)
	"Tiandeng" tunnel	**"Tiandeng" tunnel** - "Banzhulin" tunnel - "Zhengyang1#" tunnel	**Medium tunnel (649 m)** - Spacing (103 m)- Extra-long tunnel (3311 m) - Spacing (345 m)- Extra-long tunnel (3600 m)
Long tunnel	"Dawan" tunnel	"Baima" tunnel - "Yangjiao" tunnel - **"Dawan" tunnel**	ra-long tunnel (3144 m) - Spacing (76 Extra-long tunnel (6656 m) - Spacing (210 m) – **Long tunnel (2821 m)**
	"Yinpan" tunnel	"Zhongxing" tunnel - **"Yinpan" tunnel**	Extra-long tunnel (6105 m) - Spacing (27 m) – **Long tunnel (1149 m)**
	"Hujiawan" tunnel	**"Hujiawan" tunnel** - "Dongjiawan" tunnel - "Wangjiangsi" tunnel	**Long tunnel (1201 m)** - Spacing (79 m) – Long tunnel (1065 m) - Spacing (315 m) – Medium tunnel (698 m)
	"Dongjiawan" tunnel	**"Dongjiawan" tunnel** - "Wangjiangsi" tunnel	**Long tunnel (1201 m)** - Spacing (79 m) – Long tunnel (1065 m) - Spacing (315 m) – Medium tunnel (698 m)
	"Pengshui" tunnel	**"Pengshui" tunnel** - "Changtan" tunnel	**Long tunnel (2754 m)** - Spacing (485 m) - Extra-long tunnel (3215 m)
	"Shaba" tunnel	**"Shaba" tunnel** - "Shihui" tunnel - "Wuling" tunnel	**Long tunnel (2736 m)** - Spacing (299 m) - Long tunnel (2226 m)- Spacing (125 m)- Long tunnel (2414 m)
	"Shihui" tunnel	"Shaba" tunnel - **"Shihui" tunnel** - "Wuling" tunnel	Long tunnel (2736 m) - Spacing (299 m) – **Long tunnel (2226 m)** - Spacing (125 m) – Long tunnel (2414 m)
	"Dongtang" tunnel	"Tiandeng" tunnel - **"Dongtang" tunnel** - "Banzhulin" tunnel - "Zhengyang1#" tunnel	Medium tunnel (649 m) - Spacing (103 m) – **Long tunnel (1687 m)** - Spacing (160 m) - Extra-long tunnel (3311 m) Spacing (345 m) - Extra-long tunnel (3600 m)
Extra-long tunnel	"Baima" tunnel	**"Baima" tunnel** - "Yangjiao" tunnel - "Dawan" tunnel	**Extra-long tunnel (3144 m)** - Spacing (76 m) - Extra-long tunnel (6656 m) - Spacing (210 m) - Long tunnel (2821 m)
	"Yangjiao" tunnel	"Baima" tunnel - **"Yangjiao" Tunnel** -	Extra-long tunnel (3144 m) - Spacing (76 m) **Extra-long tunnel**

		"Dawan" tunnel	**(6656 m)** - Spacing (210 m) Long tunnel (2821 m)
	"Zhongxing" tunnel	**"Zhongxing" tunnel** - "Yinpan" tunnel	**Extra-long tunnel (6105 m)** - Spacing (27 m) - Long tunnel (1149 m)
	"Changtan" tunnel	"Xiatang" tunnel - **"Changtan" tunnel**	Long tunnel (2754 m) - Spacing (485 m) - **Extra-long tunnel (3215 m)**
	"Banzhulin" tunnel	"Tiandeng" tunnel - "Dongtang" tunnel - **"Banzhulin" tunnel** - "Zhengyang1#" tunnel	Medium tunnel (649 m) - Spacing (103 m) - Long tunnel (1687 m) - Spacing (160 m) - **Extra-long tunnel (3311 m)** - Spacing (345 m) - Extra-long tunnel (3600 m)
	"Zhengyang1#" tunnel	"Tiandeng" tunnel - "Dongtang" tunnel - "Banzhulin" tunnel - **"Zhengyang1#" tunnel**	Medium tunnel (649 m) - Spacing (103 m) - Long tunnel (1687 m) - Spacing (160 m) - Extra-long tunnel (3311 m) - Spacing (345 m) - **Extra-long tunnel (3600 m)**

3.2 Participants

Seven participants aged from 25 to 56 years with different driving experience were selected as test drivers. The corrected visual acuity was above 5.0, and all of them had a C1 driving license. They all performed the driver rotation test to ensure that each driver carried out at least two sets of effective tests and did not over speed. All the participants held valid driving licenses in China. All provided informed consent to participate in the test and had normal or corrected-to-normal vision.

3.3 Experimental Setup

Considering the highway driving model is given priority to small vehicles, and the collection of data using test equipment is more, need a number of experimenters and testers, the Buick GL8 automatic block commercial vehicles were selected as test vehicles as shown in Fig. 2. The eye movement data acquisition equipment was SMI2.1 glasses type eye tracker of SMI company from Germany. The sampling frequency of the eye tracker was 60 Hz, and the tracking accuracy was 0.1. The tracking ranges of the camera in the horizontal and vertical directions were 60˚ and 46˚, respectively. The vehicle speed acquisition was realized by a non-contact multi-function speed meter, having the measurement range and accuracy of 0–300 km/h and 0.1 km/h, respectively, which was used to determine whether the driver was speeding. The illuminance data acquisition equipment was the LX-9621 illuminance meter produced by Lantai company; its measurement range and accuracy were 0–50000 Lux and ±(5% + 5), respectively, and it was used to judge whether the lighting in each tunnel meets the design specifications. The whole driving process was recorded by a driving recorder with a resolution of 1920 × 1080 pixels, which was used to determine the consistency between the post-test calibration time and the road section location, as well as the driving location and the surrounding environment.

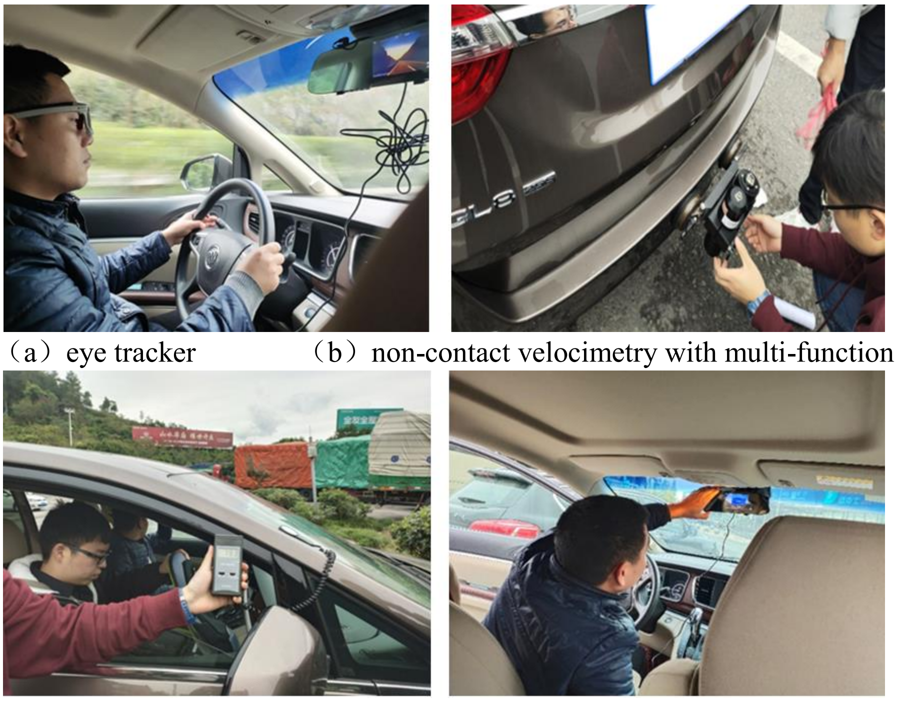

（a）eye tracker （b）non-contact velocimetry with multi-function

（c）illuminometer （d）driving recorder

Figure 2: Experimental setup

3.4 Experiment Design

In order to reduce the interference of traffic volume on the test, the real vehicle test time was from 10: 00 to 16: 00 on Tuesday to Thursday, with a small traffic volume. Since the change in the light environment outside the tunnel has a great influence on the driver, the test was carried out under good weather conditions. Before the test, the driver was informed to drive according to his driving habits, comply with traffic rules, and keep the speed in the tunnel below 80 km/h. Since passing through tunnels with different lengths has a great influence on driver's gaze behavior, according to the specification requirements, 16 single tunnels were divided into four categories according to the length, including two short tunnels with a length of less than 500 m, two medium tunnels with a length in the range of 500–1000 m, five long tunnels with a length in the range of 1000–3000 m, and four extra-long tunnels with a length of more than 3000 m [3]. The results of the same tunnel types in each tunnel group of 8 tunnel groups were compared to explore differences in drivers' fixation behaviors.

3.5 Data Collection and Analysis

To avoid the influence of environmental light change at the tunnel entrance on a driver, the eye movement data were extracted from the middle section of each tunnel using the BeGaze3.5 data analysis software. The basic types of the driver's eye movements included fixation, blinking, and saccade. The analysis of the driver's fixation duration during driving showed that approximately 99% of the single fixation durations were less than 1000 ms, so only the fixation samples shorter than 1000 ms were retained. The scan duration was from 30 ms to 120 ms, so the data within 100 ms were excluded. In the process of data processing, unclassified data, such as data on vehicle speeds exceeding 80 km/h, substandard lighting in tunnels, and fixation of over 1000 ms through a single gaze, were eliminated, and the driver's saccade data and fixation data were retained. Finally, the Markov model was applied to the

processed data to explore the law of gaze transfer of drivers and to solve the driver's fixation point in each partition. The experimental data were screened by the Pauta criterion. From the perspective of the driver's fixation transfer characteristics, the position of the fixation point at the current moment was related to only that at the previous moment. Therefore, the Markov chain was used to analyze all the schemes [36].

4. RESULTS

4.1 Gaze Time Analysis of Single Tunnel

Eighteen single tunnels were divided into four types: short tunnels, medium tunnels, long tunnels, and extra-long tunnels according to their lengths. The gaze time of drivers in each tunnel type was averaged. The gaze time of drivers in single tunnels of different lengths was obtained. The gaze time was classified and sampled at every 100 ms; the result is shown in Figure 3. In the single tunnel, the driver's gaze time was unevenly distributed in the range of 100–1000 ms, and approximately 80% of the single gaze time was less than 500 ms; the gaze time of a single short tunnel was in the range of 0–200 ms. With the increase in the single-tunnel gaze time, the fixation frequency gradually decreased. The gaze time of a single tunnel was compared with the gaze time of a tunnel with the same length in a tunnel group.

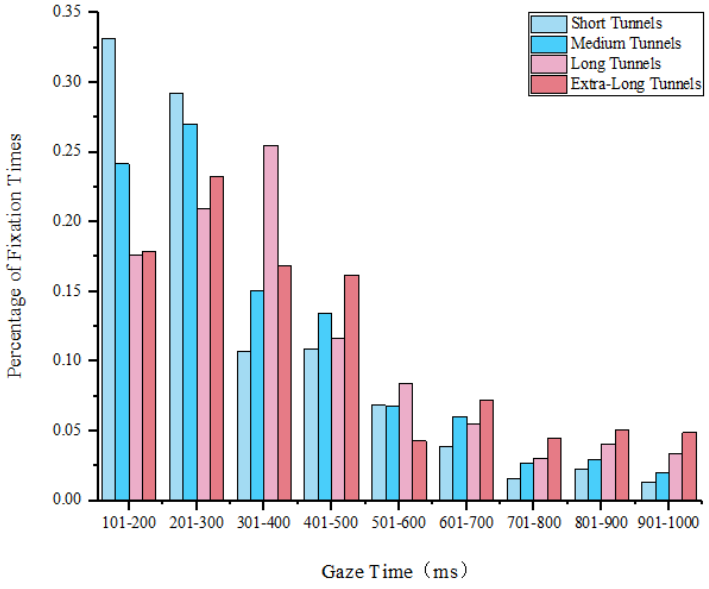

Figure 3: Single-tunnel gaze time zoning statistical chart

4.1.1 Gaze Time Analysis of Short Tunnels

The gaze times of a single tunnel and a tunnel in a tunnel group of the same length type were compared. The gaze time of a single tunnel is shown in Figure 3. The characteristic parameters of the short tunnels in the tunnel groups are given in Table 1. The comparison of the gaze time between the single short tunnel and short tunnel in the tunnel group is presented in Figure 4, where it can be seen that, due to the short tunnel length, the tunnel in the tunnel group had a smaller number of drivers' gazes but similar gaze time compared to the single

short tunnel. The gaze time of the tunnel in the tunnel group was concentrated between 200 ms and 300 ms.

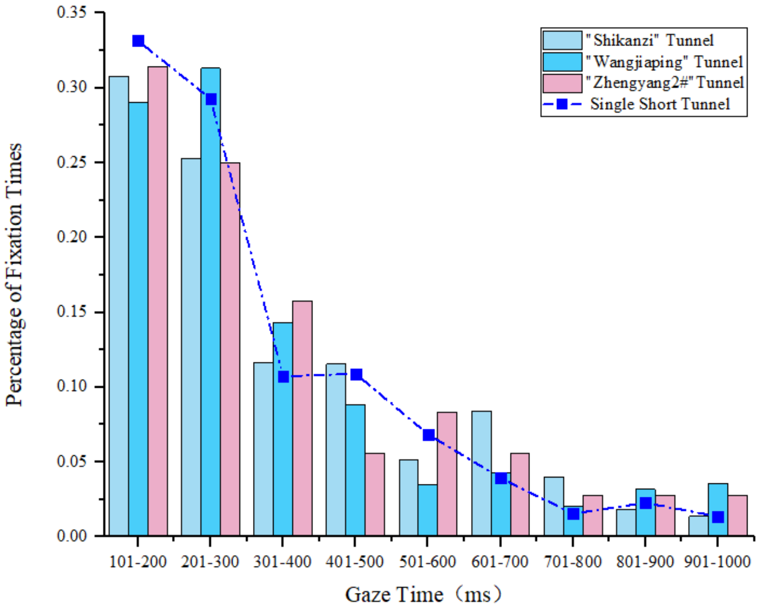

Figure 4: Statistical diagram of the gaze time of the short tunnels in the tunnel groups

4.1.2 Gaze Time Analysis of Medium Tunnels

The gaze time of the single medium tunnel was obtained from the results presented in Figure 3. The characteristic parameters of the medium tunnels in the tunnel groups are given in Table 1. The comparison of gaze time between the single tunnel and the tunnel in the tunnel group of medium length is presented in Figure 5. Compared with the short tunnel, the driver’s gaze time of the medium tunnel was concentrated below 300 ms, and the changing trend of the gaze time of the tunnel in the tunnel group was similar to that of the single tunnel. In the tunnel group, the driver’s gaze time of the medium tunnel was in the range of 700–1000 ms, which was slightly longer than that of the single medium tunnel. When the tunnel was the first tunnel in the tunnel group, its fixation time was roughly the same as that of the single tunnel.

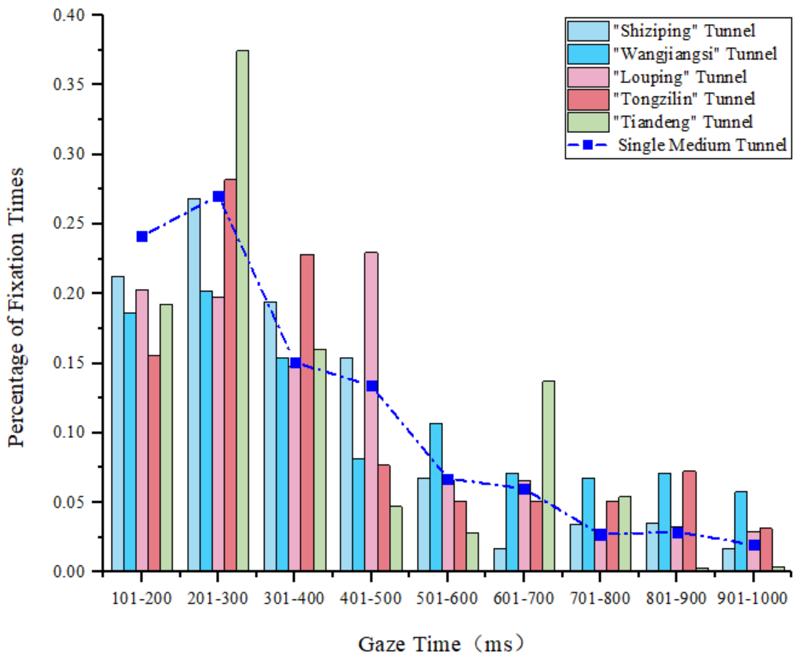

Figure 5: Statistical diagram of the gaze time of the medium tunnels in the tunnel groups

4.1.3 Gaze Time Analysis of Long Tunnels

The characteristic parameters of the long tunnels in the tunnel groups are presented in Table 1. The comparison of the gaze time between the single long tunnel and the long tunnel in the tunnel group is presented in Figure 6. Compared with the short and medium tunnels, the gaze time of the long tunnels in the tunnel groups was significantly higher than that of the single tunnel, and it was in the range of 700–1000 ms. “Dongtang” and “Dawan” tunnels were the second tunnels in the tunnel groups, and the distance between them was close to that of the upstream tunnel. However, due to the different length of the upstream tunnels, the driver’s gaze times of the two tunnels were different. “Dongjiawan” and “Shihui” tunnels were the second tunnels in the tunnel groups, and the upstream tunnels were long tunnels. However, due to the different distances from the upstream tunnel, the driver’s gaze time was longer in the “Dongjiawan” tunnel.

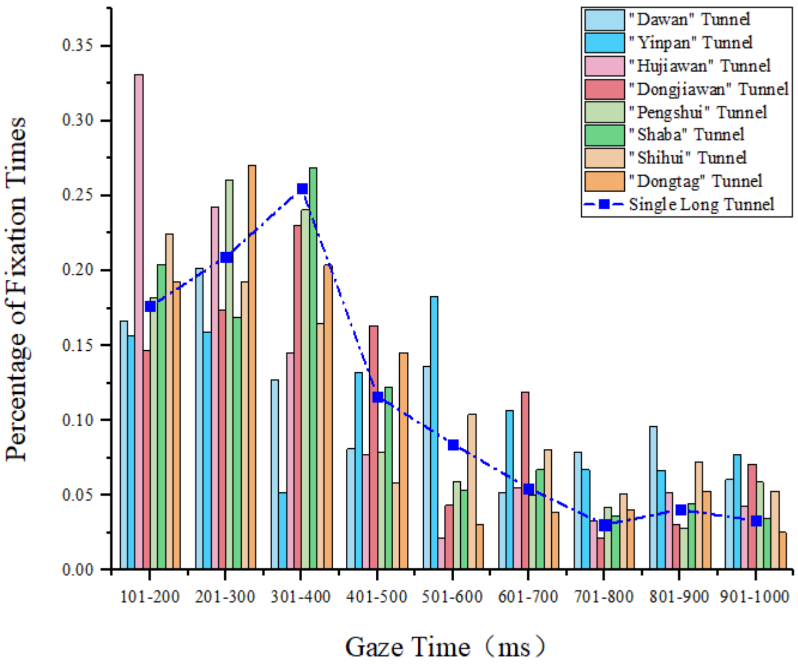

Figure 6: Statistical diagram of the gaze time of the long tunnels in the tunnel groups

4.1.4 Gaze Time Analysis of Extra-long Tunnels

The characteristic parameters of the extra-long tunnels in the tunnel groups are presented in Table 1. The comparison of the gaze time between the single extra-long tunnel and extra-long tunnel in the tunnel group is presented in Figure 7. The gaze time of the extra-long tunnel in the tunnel group was significantly longer than that of the single tunnel, and it was in the range of 600–1000 ms. “Banzhulin” and “Zhengyang1#” tunnels were the second and third tunnels in the same tunnel group, respectively, and since “Zhengyang1#” was more backward, their gaze times were in the range 800–1000 ms, which was significantly longer than those of the shorter tunnels. When the medium tunnel was the first tunnel in the tunnel group, the gaze time is roughly the same as that of the single tunnel, which is similar to that of the medium tunnel.

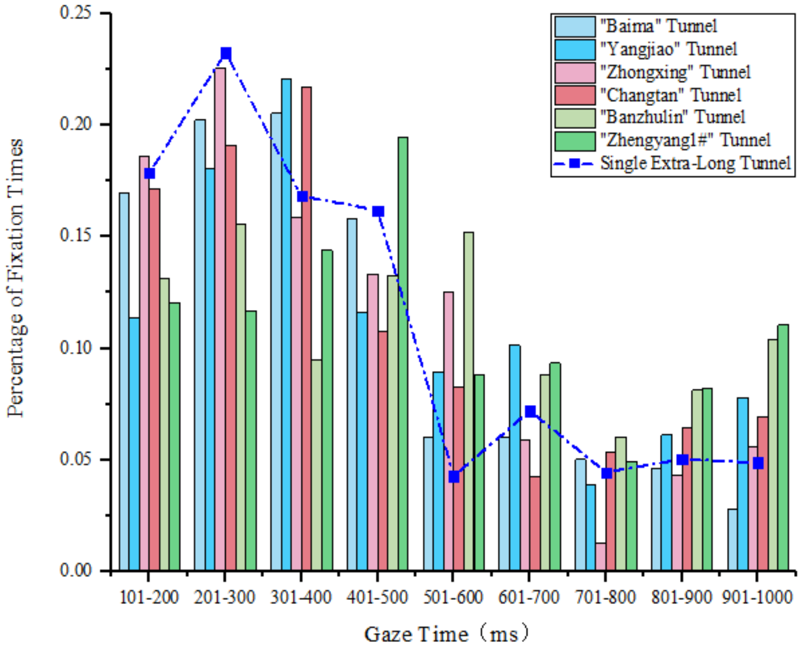

Figure 7: Statistical diagram of the gaze time of the extra-long tunnels in the tunnel groups

4.2 Gaze Area Division

To study the characteristics of driver's gaze point transfer, it was necessary to divide the driver's visual interest area first. In this study, the k-means dynamic clustering algorithm was used and combined with the driver's gaze target and driving characteristics to divide the driver's gaze interest area into five subareas, as shown in Figure 8. Area 1 was the cab area; Area 2 represented the near road section; Area 3 denoted the far ahead road section; Area 4 denoted the vehicle rearview mirrors, including left, right, and in-vehicle rearview mirrors; Area 5 represented all other areas, including tunnel vault and sidewall.

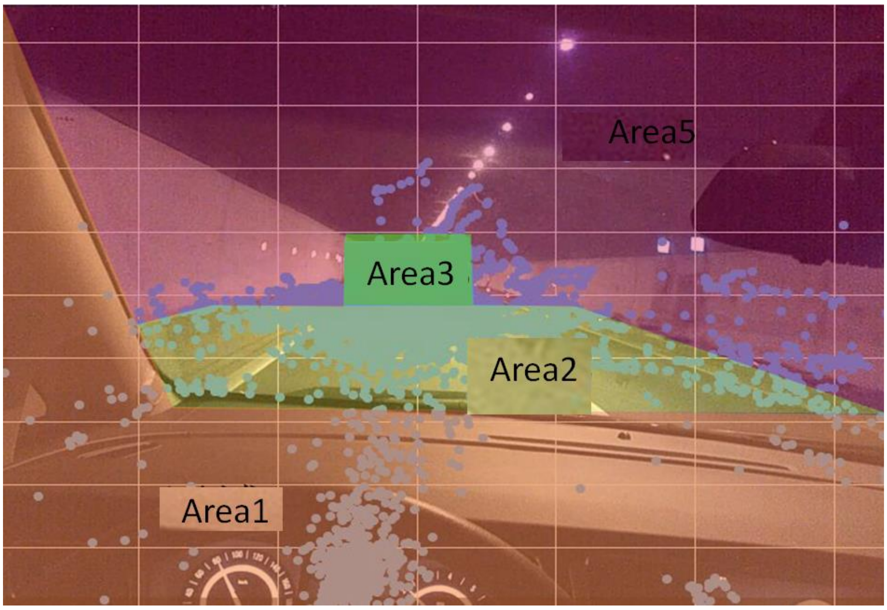

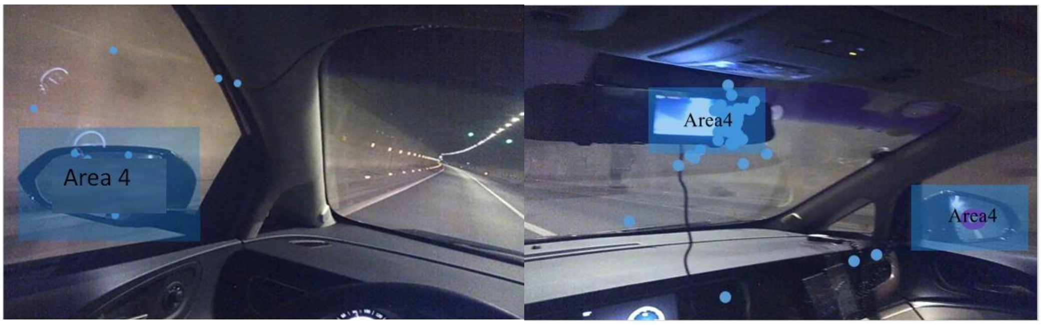

Figure: 7 Diagram of fixation area division from the driver's perspective

Aiming to pursue both left and right targets simultaneously during driving, a driver had a large deflection in the head, but the data point derived by the eye-tracker denoted a single coordinate point in a coordinate system. Therefore, to simplify the calculation, the driver's perspective was transferred into the same coordinate system.

4.3 Gaze Transfer Characteristics Analysis

Markov chain model is a random variable sequence, which can describe the state of a certain system, and the system state at a certain moment depends only on its state at the previous moment. In the driver's gaze behavior, the position of the driver's gaze point at a certain moment is related only to its position at the previous moment but not to the position of the other driver's gaze points. The driver's gaze transfer characteristics conform to the basic properties of the Markov chain. The driver's gaze Markov chain is discrete in terms of time and state. Therefore, the Markov chain is used in this work to analyze all schemes [36].

4.3.1 One-step Transition Probability Matrix of Gaze Behavior

According to the data of the eye-tracker and the division of the driver's gaze interest area, the visual attention transfer of a driver in the single tunnel was counted. After the data were processed, the Markov chain theory was used to obtain the one-step transition probability matrix of the driver's visual fixation point transformation between different regions. The one-step transition probability matrices of the single tunnels and the tunnels in the tunnel groups are presented in Tables 2. The same as in the previous analysis, tunnels were divided into four groups according to their length: short, medium, long, and extra-long.

Table 2: One-step Transition Probability Matrix of Different Type Tunnels.

Tunnel type	Area	Tunnel type	Area 1	Area 2	Area 3	Area 4	Area 5
Short tunnel	Area 1	Single short tunnel	0.8945	0.0648	0.0407	0.0000	0.0000
		Short tunnel in tunnel group	0.8712	0.0833	0.0455	0.0000	0.0000
	Area 2	Single short tunnel	0.0190	0.9360	0.0415	0.0036	0.0000
		Short tunnel in tunnel group	0.0095	0.9048	0.0857	0.0000	0.0000
	Area 3	Single short tunnel	0.0102	0.0928	0.8714	0.0000	0.0256
		Short tunnel in tunnel group	0.0041	0.0226	0.9680	0.0021	0.0032

	Area 4	Single short tunnel	0.0000	0.2500	0.0000	0.7500	0.0000
		Short tunnel in tunnel group	0.0000	0.0000	0.0000	0.8571	0.1429
	Area 5	Single short tunnel	0.1245	0.0005	0.0023	0.0000	0.8727
		Short tunnel in tunnel group	0.0000	0.1000	0.0417	0.0417	0.8166
Medium tunnel	Area 1	Single medium tunnel	0.9047	0.0277	0.0625	0.0010	0.0041
		Medium tunnel in tunnel group	0.8891	0.0453	0.0595	0.0045	0.0015
	Area 2	Single medium tunnel	0.0350	0.8950	0.0675	0.0002	0.0024
		Medium tunnel in tunnel group	0.0128	0.8312	0.1528	0.0007	0.0025
	Area 3	Single medium tunnel	0.0178	0.0476	0.9281	0.0013	0.0051
		Medium tunnel in tunnel group	0.0215	0.0347	0.9343	0.0014	0.0080
	Area 4	Single medium tunnel	0.0008	0.0270	0.0000	0.6597	0.3125
		Medium tunnel in tunnel group	0.1083	0.0639	0.0000	0.7444	0.0833
	Area 5	Single medium tunnel	0.0000	0.0336	0.0336	0.0263	0.9066
		Medium tunnel in tunnel group	0.0082	0.1227	0.0742	0.0000	0.7949
Long tunnel	Area 1	Single long tunnel	0.8926	0.0401	0.0645	0.0004	0.0024
		Long tunnel in tunnel group	0.8910	0.0202	0.0841	0.0026	0.0021
	Area 2	Single long tunnel	0.0175	0.9426	0.0328	0.0036	0.0034
		Long tunnel in tunnel group	0.0331	0.9090	0.0283	0.0086	0.0210
	Area 3	Single long tunnel	0.0125	0.0685	0.9046	0.0040	0.0103
		Long tunnel in tunnel group	0.0176	0.0200	0.9548	0.0021	0.0055
	Area 4	Single long tunnel	0.0083	0.0886	0.0083	0.8528	0.0420
		Long tunnel in tunnel group	0.0000	0.0695	0.0205	0.8372	0.0728
	Area 5	Single long tunnel	0.0357	0.0706	0.1750	0.0000	0.7187
		Long tunnel in tunnel group	0.0188	0.0353	0.0727	0.0000	0.8732
Extra-long tunnel	Area 1	Single extra-long tunnel	0.8860	0.0655	0.0400	0.0028	0.0057
		Extra-long tunnel in tunnel group	0.8929	0.0603	0.0442	0.0014	0.0012
	Area 2	Single extra-long tunnel	0.0280	0.9474	0.0183	0.0024	0.0039
		Extra-long tunnel in tunnel group	0.0144	0.9389	0.0395	0.0030	0.0041
	Area 3	Single extra-long tunnel	0.0151	0.0619	0.9122	0.0024	0.0083
		Extra-long tunnel in tunnel group	0.0132	0.0621	0.9143	0.0009	0.0094
	Area 4	Single extra-long tunnel	0.0217	0.0545	0.0961	0.7005	0.1272
		Extra-long tunnel in tunnel group	0.0000	0.0778	0.0069	0.7996	0.1157

	Area 5	Single extra-long tunnel	0.0067	0.1440	0.0463	0.0337	0.7693
		Extra-long tunnel in tunnel group	0.0196	0.1297	0.0261	0.0102	0.8144

As shown in Table 2, when the tunnel was a short or medium tunnel, the probability of repeated fixation of the driver in the same area had little difference between the single tunnels and tunnels in the tunnel groups. For long tunnels, drivers had a higher probability of repeated fixation of the same area for the tunnels in the tunnel group, and the gap was significant. The number of transfer paths was defined as a number of paths with a non-zero transfer probability. The number of fixation transfer paths in the long and extra-long tunnels was 17 and in the short and medium tunnels was 23. In the long tunnels, there were 24 and 23 gaze transfer paths for single tunnels and tunnels in the groups, respectively. In the extra-long tunnels, there was no path with a transition probability of zero for single tunnels, and there was only one path with a transition probability of zero for the tunnel groups.

4.3.2 Two-step Transition Probability Matrix of Gaze Behavior

According to the one-step transition probability matrix of a driver, the two-step transition probability matrix of the driver's gaze point was obtained. The two-step transfer calculation was performed for the five areas, and the two-step transfer probability matrices of the four tunnel types were obtained, as shown in Tables 3.

Table 3: Two-step Transition Probability Matrix of Different Type Tunnels

Tunnel type	Area	Tunnel type	Area 1	Area 2	Area 3	Area 4	Area 5
Short tunnel	Area 1	Single short tunnel	0.8030	0.1188	0.0780	0.0002	0.0000
		Short tunnel in tunnel group	0.7616	0.1448	0.0933	0.0002	0.0000
	Area 2	Single short tunnel	0.0343	0.8074	0.0698	0.0861	0.0024
		Short tunnel in tunnel group	0.0166	0.8241	0.1588	0.0000	0.0006
	Area 3	Single short tunnel	0.0246	0.1535	0.7816	0.0001	0.0402
		Short tunnel in tunnel group	0.0081	0.0451	0.9397	0.0042	0.0031
	Area 4	Single short tunnel	0.0018	0.4286	0.0054	0.5642	0.0000
		Short tunnel in tunnel group	0.0000	0.0000	0.0119	0.7511	0.2370
	Area 5	Single short tunnel	0.2205	0.0139	0.0000	0.0000	0.7656
		Short tunnel in tunnel group	0.0051	0.1824	0.1187	0.0346	0.6592
Medium tunnel	Area	Tunnel type	Area 1	Area 2	Area 3	Area 4	Area 5
	Area 1	Single medium tunnel	0.8221	0.0537	0.1161	0.0001	0.0080
		Medium tunnel in tunnel group	0.7949	0.0820	0.1134	0.0067	0.0030
	Area 2	Single medium tunnel	0.0631	0.8072	0.1244	0.0003	0.0050
		Medium tunnel in tunnel group	0.0262	0.7021	0.2648	0.0013	0.0056

	Area 3	Single medium tunnel	0.0337	0.0882	0.8657	0.0021	0.0103
		Medium tunnel in tunnel group	0.0401	0.0638	0.8793	0.0027	0.0141
	Area 4	Single medium tunnel	0.0003	0.0629	0.0128	0.4664	0.4575
		Medium tunnel in tunnel group	0.1579	0.1073	0.0226	0.5816	0.1307
	Area 5	Single medium tunnel	0.0019	0.0631	0.0644	0.0452	0.8255
		Medium tunnel in tunnel group	0.0194	0.0969	0.0300	0.0003	0.8534
Long tunnel	Area 1	Single long tunnel	0.7990	0.0763	0.1191	0.0010	0.0046
		Long tunnel in tunnel group	0.7962	0.0380	0.1564	0.0045	0.0049
	Area 2	Single long tunnel	0.0327	0.8919	0.0627	0.0063	0.0064
		Long tunnel in tunnel group	0.0578	0.8456	0.0447	0.0147	0.0372
	Area 3	Single long tunnel	0.0243	0.1252	0.8262	0.0072	0.0171
		Long tunnel in tunnel group	0.0331	0.0375	0.9150	0.0038	0.0106
	Area 4	Single long tunnel	0.0180	0.1598	0.0259	0.7290	0.0673
		Long tunnel in tunnel group	0.0030	0.1157	0.0555	0.7987	0.0271
	Area 5	Single long tunnel	0.0524	0.1264	0.2731	0.0009	0.5472
		Long tunnel in tunnel group	0.0174	0.0638	0.1340	0.0163	0.7685
	Area	Tunnel Type	Area 1	Area 2	Area 3	Area 4	Area 5
	Area 1	Single extra-long tunnel	0.7873	0.1229	0.0736	0.0052	0.0110
		Extra-long tunnel in tunnel group	0.7993	0.1125	0.0833	0.0025	0.0024
Extra-long tunnel	Area 2	Single extra-long tunnel	0.0510	0.9012	0.0356	0.0046	0.0076
		Extra-long tunnel in tunnel group	0.0270	0.8854	0.0751	0.0054	0.0071
	Area 3	Single extra-long tunnel	0.0286	0.1162	0.8351	0.0045	0.0156
		Extra-long tunnel in tunnel group	0.0248	0.0160	0.9420	0.0021	0.0151
	Area 4	Single extra-long tunnel	0.0386	0.1103	0.0156	0.6726	0.1629
		Extra-long tunnel in tunnel group	0.0031	0.1790	0.0241	0.7028	0.0909
	Area 5	Single extra-long tunnel	0.0152	0.2380	0.0828	0.0465	0.6176
		Extra-long tunnel in tunnel group	0.0349	0.1177	0.1162	0.0158	0.7154

The numbers of driver's gaze transfer paths in the single short tunnels and short tunnels in the groups were 21. In tunnels of the other lengths, there was no path with a transition probability of zero. If the initial position of a fixation point falls in the same region, that is called "looking back" phenomenon after two transfer steps. The two-step transition probability of the driver's gaze in this region is the maximum, and it is higher than those of the other regions. The probability of the driver looking back at the same area in the tunnels in the tunnel groups was slightly higher than that in the single tunnels. With the increase in the

tunnel length, the probability of looking back in areas 2 and 3 increased, and in the extra-long tunnels in the tunnel groups, this probability reached 0.9420.

4.3.3 Stable Distribution of Gaze Behavior

The stationary distribution probabilities of different tunnel types were calculated.

$$\pi_{Single\ short\ tunnel}=\begin{bmatrix}0.1648\\0.4048\\0.4007\\0.0061\\0.0236\end{bmatrix}\ \pi_{Single\ medium\ tunnel}=\begin{bmatrix}0.1542\\0.3320\\0.4388\\0.0102\\0.0648\end{bmatrix}\ \pi_{Single\ long\ tunnel}=\begin{bmatrix}0.1403\\0.4085\\0.4030\\0.0170\\0.0312\end{bmatrix}\ \pi_{Single\ extra\text{-}long\ tunnel}=\begin{bmatrix}0.1317\\0.4883\\0.2953\\0.0205\\0.0642\end{bmatrix}$$

$$\pi_{Short\ tunnel\ in\ tunnel\ groups}=\begin{bmatrix}0.0578\\0.1847\\0.6891\\0.0399\\0.0286\end{bmatrix}\ \pi_{Medium\ tunnel\ in\ tunnel\ groups}=\begin{bmatrix}0.1788\\0.2127\\0.5430\\0.0112\\0.0543\end{bmatrix}$$

$$\pi_{Long\ tunnel\ in\ tunnel\ groups}=\begin{bmatrix}0.1427\\0.1786\\0.6034\\0.0191\\0.0562\end{bmatrix}\ \pi_{Extra-long\ tunnel\ in\ tunnel\ groups}=\begin{bmatrix}0.1237\\0.3904\\0.4415\\0.0125\\0.0319\end{bmatrix}$$

Regardless of the tunnel type (single tunnel or tunnel in a group), drivers paid the most attention to areas 2 and 3. With the increase in the tunnel length, the driver’s attention to these two areas increased, and it was higher for the tunnels in the groups than for the single tunnels.

5. DISCUSSION

This study analyzes the difference between mountainous-expressway single tunnels and tunnel groups based on eye gaze behavior. Compared with the single tunnel, the fixation time of drivers in the tunnel groups with the same length was longer, and it was in the range of 700–1000 ms. This indicated that the driver’s visual load increased, and it took longer to identify objects in the tunnel. With the increase in the tunnel length, the frequency of the driver’s concentrated gaze showed an upward trend, from 200 ms to 400 ms, which also indicated that the driver’s psychological pressure in the tunnel group increased. When the tunnel length was the same, the driver’s position in the tunnel group was more backward, and the fixation duration was longer. The reason was that the driver had experienced multiple tunnels, so the cumulative visual effect increased. However, this was related to the tunnel position in the tunnel group. If the interval between tunnels was short, the driver could not adapt to the external environment of the tunnel. The frequent “black hole” and “white hole” effects led to a longer time needed to identify objects in the downstream tunnel. In addition, the length of the upstream tunnel also affected the driving safety of the downstream tunnel in the tunnel group. The longer the upstream tunnel length, the longer the driver’s gaze duration in the downstream tunnel.

As shown in Tables 2, in long and extra-long tunnels, the probability of driver’s repeated fixation in the same area was significantly different for single tunnels and tunnels in the

group. The results showed that it was more difficult for drivers to obtain information in long and extra-long tunnels than in medium and short tunnels, and the probability of repeated gaze on the target was higher. In the short tunnels, the gaze transfer paths were short because the tunnel length was short, and the driving time through the tunnel was also short, so the scope of driver's attention was limited. Two-step gaze transition probability can reflect a continuous transition behavior. As shown in Tables 3, the driver gazed at the short transition path in the short tunnel because this behavior was the reflection of the one-step transition probability was the same as the one-step transition probability. The driver's two-step transition probability of looking back to the same region was the largest, which was related to the specific environment of a single tunnel. The two-step transition probability of drivers in the tunnel groups was larger than that in the single tunnels, indicating that the drivers needed to confirm the region repeatedly. With the increase in the tunnel length, the two-step transition probabilities of drivers in areas 2 and 3 showed an upward trend, and this increase in the tunnel groups was more significant. The two-step transition probability in the extra-long tunnels in the tunnel groups was 0.9420, which can be explained as follows. Area 3 included the tunnel entrance, which is far ahead, indicating that the driver had been looking for the tunnel exit and was anxious to leave the tunnel. In the stationary distribution of the gaze behavior, with the increase in the tunnel length, the driver's attention to areas 2 and 3 increased, and it was higher for the tunnel group than for the single tunnel. However, the driver paid too much attention to these two areas, which led to the neglect of the other areas. However, the lack of attention to traffic speed and surrounding traffic conditions is prone to visual fatigue and safety hazards, which can cause traffic accidents.

In general, when the tunnel length was shorter than 1000 m, which included short and medium tunnels, there were little differences in the fixation time and fixation point transfer between the single tunnels and the tunnel groups; thus, in this case, the safety of the tunnel groups does not require special consideration. When the tunnel length was longer than 1000 m, which included long and extra-long tunnels, the repeated fixation probability and two-step transition probability of drivers in the tunnel groups were higher than those in the single tunnels, which indicated that the special environment of the tunnel group had a great influence on the driver's fixation characteristics. Thus, for these tunnels, it is necessary to pay special attention to driving safety, and the design and management methods of single tunnels cannot be applied directly. Particularly, for extra-long tunnels, which were located in the back of the tunnel groups, the length of the upstream tunnel was long, and the distance between the upstream tunnel and the tunnel was short; thus, the driver was passing through multiple tunnels continuously, driving in tunnels for a long time, and frequently experiencing the effect of black and white holes, so the cumulative visual pressure could not be effectively released. Considering that the superposition effect of psychology and vision can easily increase the driving risk, the design and management of these tunnels require further research and analysis.

6. CONCLUSION

To analyze the difference between the tunnel groups and single tunnels in terms of operational safety, this paper conducts a real vehicle test. The test results show that when the tunnel length is short or medium, there are little differences in the fixation time and fixation point transfer between the two tunnel types, and the safety of the tunnel groups does not require special consideration. When the tunnel length is longer than 1000 m, the repetition fixation probability and two-step transition probability of drivers in the tunnel groups are higher than those in the single tunnels; thus, for these tunnels, it is necessary to consider

driving safety further. Particularly, for extra-long tunnels located at the back of the tunnel groups, the design and management of these tunnels require special attention. The methods and techniques for improving the driving safety of single tunnels have been widely studied, but there have been fewer studies on mountain expressway tunnels. Therefore, this study can provide a helpful reference for understanding the difference in driving safety between single tunnels and tunnels in a group. The presented research results can also provide a theoretical basis for the design and management of long and extra-long tunnels in the mountainous expressway. The main limitation of this study is that it does not consider the influence of tunnel alignment on a driver’s visual characteristics. The curve section in tunnel groups could be considered in future work.

ACKNOWLEDGMENTS

This work was supported by the Science and Technology Bureau Foundation and Frontier Project of Chongqing (cstc2019jcyj-msxmX0695), Science and Technology Research Program of Chongqing Municipal Education Commission (Grant No. KJQN201900722).

AUTHOR CONTRIBUTIONS

The authors confirm contribution to the paper as follows: study conception and design: Ting. Shang, Hongjiao. Qi, Tangzhi. Liu, and An. Huang; data collection: Hongjiao. Qi, and An. Huang; analysis and interpretation of results: Ting. Shang, and Tangzhi. Liu; draft manuscript preparation: Ting. Shang, and Hongjiao. Qi. All authors reviewed the results and approved the final version of the manuscript.

References

[1] 胡顺峰. 山区高速公路隧道群交通事故特征及致因机理分析[J]. 公路, 2016(5):134-138.

HU Shun-feng, “Traffic Accident Characteristics and Mechanism Analysis of Mountain Highway Tunnel Group,” *Highway*, vol. 171, no. 5, pp. 134-138, 2016.

[2] Design specification for highway alignment, JTG D20-2017.

[3] Specifications for design of highway tunnel, JTG 3370.1-2018.

[4] Road traffic signs and markings, GB5768.2-2009.

[5] Guidelines for design of highway grade-separated intersections, JTG/T D21-2014.

[6] Verwey, W. B., “Effects of Tunnel Entrances on Driver's Physiological Condition and Performance,” Presented at the Transportation Research Board, Washington, D.C., 1995.

[7] Qi, W., B. Shen, and L. Wang, “Model of Driver's Eye Movement and ECG Index Under Tunnel Environment Based on Spatiotemporal Data,” *Journal of Advanced Transportation*, no.3, pp. 1-1, 2020.

[8] Yang, Y., and F. Chen, “Research on the Characteristics of Driver Visual Behavior in Highway Extra-Long Tunnel,” *Presented at IOP Conference Series Earth and Environmental Science, Kuala Lumpur, MALAYSIA*, 2019.

[9] Ye,F., K. Ying, E. Su, Y. Wei, and X. Liang, “Comfort and Safety Evaluation of Tunnel Portal Landscape Design Based on Eye Movement Index,” *Journal of Tongi Universit*, vol. 49,no. 2, pp. 218-226.

[10] He,C., B. Chen, J. Wang, and Y. Shi, “Research on Allocation of the Driver's Attention on the Tunnel Sections of A Mountain Freeway,” *In Proceedings of the 10th Internationa Conference of Chinese Transportation Prfessioals*, no 382, pp. 444-452, 2010.

[11] He,C., B. Chen, J. Wang, and Y. Shi, “Research on Allocation of the Driver's Attention on the Tunne Du, Z., and X. Pan. Application Research of Visual Cognition Probabilistic Model on Urban Tunnel's Sign,” *Presented at Intenationa Coferene on Measuring Techology and Mechatronics Automation, Zhangjiajie, Hunan, China*, no 3, pp. 490-493, 2009.

[12] Shang, T., H. Lu, P. Wu, and X. Lu, “Method of Setting Exit Advance Guide Signs in Highway Tunnels Based on the Driver's Eye Movement with Markov Chains,” *IEEE Access*, no 9, pp. 24079-24092, 2021.

[13] Bai, H., L. Yu, T. Zhang, D. Xie, and Z. Liang, “Locating Speed Limit Signs for Freeway Tunnel Entrance and Exit,” *Intermatonal Conference on Transportation and Development 2018: Connected and Autonomous Vehicles and Transportation Safety - Selected Papers from the International Conference on Transportation and Development, Pittsburgh, Pennsylvania*, pp. 377-386, 2018.

[14] Pan, X., Z. Du, and Z. Yang, “Relationship Between Eye Movement Index and Horizontal Alignment at Entrance of Highway Tunnel,” *Journal of Tongji University*, vol. 36, no. 12, pp. 1661-1664, 2008.

[15] Wang S., Z. Du, G. Chen, H. Zheng, Z. Tang, and F. Jiao, “Drivers’ Visual Characteristics in Small-Radius Optically Long Tunnels on Rural Roads,” *Tunnelling and Underground Space Technology*, vol. 113, no. 3, pp. 103969, 2021.

[16] Tang, L., X. Zhuo, X. Yao, J. Wang, and L. Xiu, “Research on Color Combination of Colored Pavements in Tunnels Based on Indexes of Lighting and Eye Movement,” *In Proceedings of the 19th COTA International Conference of Transportation Prfessonals*, pp. 3724-3735, 2019.

[17] Huang, Z., J. Xu, J. X. Qin, Y. Liu, and Q. Sheng, “Study on the Influence of Anti-Slipping Layer Color on Driving Safety at the Tunnel Entrance,” *Presented at IOP Conference Seriese: Earth and Environmental Science*, vol. 526, no. 1, pp. 012214, 2020.

[18] Wang, J., P. Amjad, Z. Wang, C. Han, and L. Hu, “Crash Analysis of Chinese Freeway Tunnel Groups Using a Five-Zone Analytic Approach,” *Tunnelling and Underground Space Technology*, vol. 82, pp 358-365, 2018.

[19] Xiong, X., H. Wang, T. Shang, and S. Tian, “Construction of Accident Rate Model for Tunnel Group Sections of Expressway in Mountainous Areas,” *Presented at 3rd Internati-onal Conference on Traffic Engineering and Transportation System*, Jiaozuo, 2019.

[20] Cao, H., B. Chen, J. B. Wang, and Y. Shi, “Research on Allocation of the Driver's Attention on the Tunnel Sections of a Mountain Freeway,” *Presented at Tenth International Conference of Chinese Transportation Professionals (ICCTP)*, 2010.

[21] Yan, B., H. Chen, and L, “Wang. Visual Characteristics of the Driver to Tunnel Group Traffif Safety,” *In Proceedings of the 3rd Intemational Conference on Transportation Engineering*, pp. 3009-3014, 2011.

[22] Yan, B., C. Li, and B. Zhao, “Perceptual Characteristics of Driver to the Tunnel Group in Traffic Safety,” *Intemnational Coferene on Remote Sensing, Enwironment and Transportation Engineering*, pp. 4253-4256, 2011.

[23] Li, X., J. Zhang, and P. Wang, “Cooperative Control Technology of Electromechanical Facilities Under Emergencies in Freeway Tunnel Group,” *Presented at 4th International Conference on Electromechanical Control Technology and Transportation (ICECTT)*, 2019.

[24] Guo, C., M. Wang, and L. Yang, “Intelligent Traffic Safety Control Technology of Highway Adjoining Tunnel and Tunnel Group,” *Presented at International Conference on Transportation Engineering (ICTE)*, 2013.

[25] Jiang, F., Y. Fang, C. Zhou, and X. Zhang, “Intelligent Control of Ventilation System for Tunnel Group of Highway Section,” *Applied Mechanics and Materials, Trans Tech Publications Ltd*, 2014.

[26] Shiferaw, B., L. Downey, and D. Crewther, “A Review of Gaze Entropy as A Measure of Visual Scanning Efficiency,” *Neuroscience & Biobehavioral Reviews*, vol. 96, pp. 353-366, 2019.

[27] Oliver, N., and A.P. Pentland, “Graphical Models for Driver Behavior Recognition in Asmartcar,” *In Proceedings of the IEEE intelligent vehicles symposium*, pp. 7-12, 2000.

[28] de La Bourdonnaye, F., R. Setchi, and C. Zanni-Merk, “Gaze Trajectory Prediction in the Context of Social Robotics,” *IFAC-PapersOnLine*, vol. 49, no. 19, pp. 126-131, 2016.

[29] Chen, Z., S. Zhang, S. Mcclean, G. Lightbody, and A. Garifullina, “Using Eye Tracking to Gain Insight Into TV Customer Experience by Markov Modelling,” *IEEE SmartWorld, UIC*, pp.916-921, 2019.

[30] Zhang, S., S. Mcclean, A. Garifullina, I. Kegel, and B. Scotney, “Evaluation of the TV Customer Experience Using Eye Tracking Technology,” *Presented at British HCI Conference, British Computer Society, Bournemouth University, U.K.*, 2018.

[31] Chatterjee, M., N. V. Manyakov, A. Bangerter, D. A, Kaliukhovich, G. Pandina. Learning Scan Paths of Eye Movement in Autism Spectrum Disorder,” *Studies in Hwelth Technology and Informatics*, vol. 270, pp. 287-291, 2020

[32] Mauricio M., R. bryan, L. joonbum, B. Mehler, and L. Fridman, “Distinguishing Patterns in Drivers’ Visual Attention Allocation Using Hidden Markov Models,” *Transportation Research Part F: Traffic Psychology and Behaviour*, vol. 43, pp. 90-103, 2016.

[33] Reimer, B., B. Mehler, M. Muñoz, J. Dobres, D. Kidd, and I. Reagan, “Patterns in Transitions of Visual Attention During Baseline Driving and During Interaction with Visual-Manual and Voice-Based Interfaces,” *Ergonomics, (just-accepted)*, pp. 1-34, 2021

[34] Tian, L., and J. Jiang, “Traffic Safety of Mountainous Expressway Tunnel Based on Speed Cpntrol,” *International Journal of Simulation: Systems, Science and Technology*, vol. 17, pp. 10, 2016.

[35] Zhan, W., Q. Lü, and Y. Shang, “Analysis of Gray-Markov Forecasting for Traffic Accidents in Highway Tunnel Group Region,” *Journal of Jilin University (Engineering and Technology Edition)*, vol. 44, pp. 62-67, 2014

[36] Li, Y.; Wang, F.; Ke, H.; Wang, L.L.; Xu, C.C, “A Driver’s Physiology Sensor-Based Driving Risk Prediction Method for Lane-Changing Process Using Hidden Markov Model,” *Sensors*, vol. 19, pp. 2670, 2019.

Click here to access/download
Supporting Information
Support data.pdf
