# Supplementary material for: A comparative driving safety study of mountainous expressway individual tunnel and tunnel group based on eye gaze behavior
Source: PLoS One. 2022 Feb 14;17(2):e0263835. doi: 10.1371/journal.pone.0263835 (PMC8843135; doi:10.1371/journal.pone.0263835)
Supplement: Attachment — Submitted filename: Response to Reviewers.docx [file pone.0263835.s003.docx]

**Response to Reviewers**

Dear Editor and Reviewers:

Thank you for your letter and reviewers’ comments concerning our manuscript entitled “A Comparative Driving Safety Study of Mountainous Expressway Individual Tunnel and Tunnel Group Based on Eye Gaze Behavior”. Those comments are all valuable and very helpful for revising and improving our paper, as well as the important guiding significance to our researches. We have studied your letter, and revised the manuscript which we hope meet with approval. Here below is our description on revision according to journal requirements and the reviewers’ comments.

According to journal requirements, we make the following statement. Firstly, we have modified the manuscript style templates to meet PLOS ONE's style requirements. Secondly, this work was supported by the Science and Technology Bureau Foundation and Frontier Project of Chongqing (cstc2019jcyj-msxmX0695), Science and Technology Research Program of Chongqing Municipal Education Commission (Grant No. KJQN201900722), National Natural Science Foundation of China (Grant No.52172341)), and there was no additional external funding received for this study, please refer to Annex 1 for the Funding Statement. Thirdly, the authors have declared that no competing interests exist. The above two points have been modified in the cover letter. The fourth, as for the use of pictures in the paper, we have removed Figure 1 from the manuscript and uploaded the copy of Figure 8(Figure 7 in the revised manuscript) see Annex 2 and. The fifth, we have uploaded the documents of consent form related to Figure 2(Figure 1 in the revised manuscript), as shown in Annex 3. In addition, we have removed any funding-related text from the manuscript and added caption for Supporting Information files at the end of the manuscript. Finally, we have reviewed our reference list to ensure that it is complete and correct.

The main correction in the paper and the responds to the reviewer’s comment are as flowing:

Response to the reviewer’s comments:

Reviewer #1:

1. It is better to specify the content of “suitable design”, just a brief presentation, which could be provide an information about the relationship between eye gaze characteristic and “which part of the design”.

**Response:** Thank you very much for your suggestion. The analysis of driver's eye gaze characteristics, such as gaze time and gaze area division are to provide data basis for gaze probability transition in section 4.3.

2. Introduction line 44-48 on page 2 “The cumulative visual superposition effect is obvious, which causes an excessive visual load to drivers and can easily cause traffic accidents due to uncontrolled operation.” Is there any study supporting this claim?

**Response:** Thank you very much for your questions. Yes, there are studies to support this claim. For example, Duan Mengmeng put forward the concept of " load repeat accumulation effect" to express the phenomenon of visual changes of drivers in the process of frequent access to tunnels in his research on *Drive’s Visual Load at Tunnel Entrance and Exit of Sections with High Ratio of Tunnels*, and pointed out that this phenomenon causes an excessive visual load to drivers and is extremely detrimental to driving safety. The " load repeat accumulation effect" proposed above is the similar concept as "The cumulative visual superposition effect " mentioned in this paper.

[1] Duan, M. M, Tang, B. M., Hu X. H., etc. “Research on driver's visual load at tunnel entrance and exit of high tunnel ratio section”. *Transportation system engineering and information*, vol. 18, pp. 113-119, 2018.

3. The author often discusses about the difference of the gaze transition probability between individual tunnel and tunnel group but it is better to test the difference statistically (statistically significant or not).

**Response:** Thank you very much for your suggestion. Your suggestion that the use of statistical significance test is a good way to further validate the difference between single tunnel and tunnel group driver’s fixation transfer. From the one-step transfer matrix, two-step transfer matrix and steady-state matrix calculated by the Markov theory in this paper, it can be seen that the probability values of the single tunnel and tunnel group in the short, medium, long and extra-long four types of driver’s fixation points to different regions are distributed between 0～1, and the difference is not significant. The sample size is small, and the proportion of ‘0’ value in the one-step, two-step and steady-state matrix is high, so it is not suitable for the analysis of statistical significance test. In addition, we reviewed the relevant literature and found that scholars used Markov theory to study the driver's fixation transfer characteristics did not use statistical difference test for comparative analysis[2][3]. However, the suggestions you put forward are very meaningful, and we will continue to explore the methods that can analyze the difference of the driver’s fixation transfer distribution in single tunnels and tunnel groups in subsequent studies.

[2] Peng, J. S., Wang, C. W., Fu, R., etc. “Extraction of parameters for lane change intention based on driver's gaze transfer characteristics”. *Safety Science*, vol. 126 :104647.

[3] Guo, TI, Pan Shu, Shao Fei, etc. “Characteristics of urban tunnel driver's gaze behavior”. *Journal of Southeast University (English Edition)*, vol. 37, pp. 325-331, 2021.

4. It is good to mention the power of the Markov model used.

**Response:** Thank you very much for your questions. Markov model has typical characteristics of stochastic process without aftereffect. To determine the state of the model at “T+1”, we just need to know its state at “T”, and do not need to know its previous state. Similarly, the driver in the driving process, the next fixation in the region of interest is only related to the current state, and has nothing to do with the previous fixation. If the sweep behavior is removed, all the fixation is discrete in time and state. Therefore, the use of discrete Markov chain theory can solve the problem well.

5. In the discussion part, please discuss published studies that show similar or dissimilar trends should be discussed.

**Response:** Thank you very much for your suggestion. We have discussed the similar trends between this paper and published studies in the discussion section. Please refer to section 5 of the file named ‘Revised Manuscript with Track Changes’ for details.

6. Please add possible ideas for further research in the future, to line 446-448 on page 20. Elaborate, not only mention the curve section.

**Response:** Thank you very much for your suggestion. We have added possible ideas for further research in the future. We have marked the corrections in red in the manuscript. Please refer to section 6 of the file named ‘Revised Manuscript with Track Changes’ for details.

Reviewer #2:

1. Please explain why a 500m interval between two adjacent tunnel is used as threshold for defining tunnel group?

**Response：**Thank you very much for your questions. According to the early research group, we define the tunnel group from the perspective of the driver's visual characteristics. As shown in Fig 1：

Fig 1. Intersection spacing diagram of tunnel connection

***S=S_1_+S_2_***	(1)

*S_1_* is the clear adaptation distance at the exit of the previous tunnel; *S_2_* is the gaze distance from the driver’s gaze point at the distance between the two tunnels to the entrance of the descendant tunnel. When the driver is driving in the adjacent tunnel and the continuous tunnel, the former tunnel will affect the driving of the latter tunnel. The tunnel group is defined as the length of the middle segment of the continuous tunnel.

①Light adaptation distance *S_1_*

The driver will experience a clear adaptation process when driving out of the tunnel during the daytime, which will make the driver difficult to recognize. Studies in China and abroad suggest that the clear adaptation time is short, and the driver needs 1～3 seconds to gradually adapt. Considering the driving safety of the driver, this paper takes the apparent adaptation time of the driver out of the first tunnel as 3 seconds, and the speed limit of the tunnel group section is 80 km / h, and calculates that the apparent adaptation distance S1 required by the driver after driving out of the tunnel is about 67 m.

②Fixation distance *S_2_*

The relationship between the driver's gaze distribution and running speed under normal conditions, as shown in Table 1, curve fitting of the given data, the formula (2) is obtained.

Table 1 Driver's gaze distribution and speed

| Vehicle speed（km/h）  Fixation distance（m） | 40 | 72 | 85 | 105 |
| --- | --- | --- | --- | --- |
|  | 190 | 360 | 500 | 600 |

$\text{y}=6.4689x-75.9$ (2)

R^2^=0.9828，and the fitting degree is good.

According to Formula (2), the driver’s visual distance is 442 m when the speed limit of mountainous high-speed tunnel group is 80 km / h.

To sum up, considering the driver's visual characteristics, the mountain highway tunnel group section is defined as two or more tunnels, and the tunnel spacing is less than **500 m** continuous tunnel. The tunnel group with tunnel spacing less than 70 m (S1) is called adjacent tunnel group, and the tunnel group with tunnel spacing more than 70 m is called continuous tunnel group.

2. The definitions for short, medium, long and extra-long tunnels can be moved from "3.4 Experiment Design, L222~225" upward like "3.1 Single Tunnel and Tunnel Group Experiments", for an easier understanding.

**Response:** Thank you very much for your suggestion. We have correction according to the Reviewer’s suggestion. Please refer to ‘Revised Manuscript with Track Changes’ for details.

3. L351, The two-step transfer, or The two-step transition? which word is better? like say, in the whole paper, are "transfer" and "transition" the same? could be replaceable? or used in the fixed terms each other? Please note clearly.

**Response:** We looked through a lot of relevant references and found that “transition” is the most popular word for the “Markov transition probability”. Therefore, "The two-step transition" is used in this manuscript. In order to ensure the consistency of the words used throughout the manuscript, about “Markov transition”, we all use “transition”. In addition, “transfer” is commonly used in the “fixation point transfer; gaze transfer; visual transfer, etc”. We have marked the corrections in red in the manuscript. Please refer to ‘Revised Manuscript with Track Changes’ for details.

4. L437 "repetition" should be revised as "repeated".

**Response:** Thank you very much for your suggestion, we have revised the text according to the reviewer 's suggestion. Please refer to the file named ‘Revised Manuscript with Track Changes’ for details.

5. Some comments are marked in the original paper.

**Response:** Thank you very much for your suggestion. We modified the article according to your comments that are marked in the original paper. Please refer to ‘Revised Manuscript with Track Changes’ for details.

Special thanks to you for your good comments.

We tried our best to improve the manuscript and made some change in the manuscript. These changes will not influence the content and framework of the paper. And here we did not list the changes but marked in red in the file named ' Revised Manuscript with Track Changes '.

We appreciate for Editor and Reviewers’ warm work earnestly, and hope that the correction will meet with approval.

Once again, thank you very much for your comments and suggestion.
